# Trends in cause-specific mortality among persons with Alzheimer’s disease in South Carolina: 2014 to 2019

**DOI:** 10.3389/fnagi.2024.1387082

**Published:** 2024-04-17

**Authors:** Candace S. Brown, Xi Ning, Amy Money, Mauriah Alford, Yinghao Pan, Margaret Miller, Matthew Lohman

**Affiliations:** ^1^Department of Public Health Sciences, University of North Carolina, Charlotte, Charlotte, NC, United States; ^2^Department of Statistics, Colby College, Waterville, ME, United States; ^3^Department of Health and Human Services, University of North Carolina at Wilmington, Wilmington, Wilmington, NC, United States; ^4^Department of Mathematics and Statistics, University of North Carolina, Charlotte, Charlotte, NC, United States; ^5^Arnold School of Public Health, University of South Carolina, Columbia, SC, United States

**Keywords:** survival analysis, death certificates, dementia, underlying causes, Alzheimer’s disease

## Abstract

**Introduction:**

Inconsistencies of reports contributes to the underreporting of Alzheimer’s disease (AD) on death certificates. Whether underreporting exists within South Carolina has not been studied.

**Methods:**

We conducted a prospective, population-based study on a cohort of persons (*N* = 78,534) previously diagnosed with AD and died between 2014–2019. We linked vital records with the South Carolina Alzheimer’s Disease and Related Dementias Registry to investigate their cause of death and survival rates. Descriptive analyses calculated frequencies of demographic and health-related characteristics. Turnbull’s method estimated the survival probabilities for different subgroups of patients. Hazard ratios were computed from the Cox proportional hazards model, adjusting for the following confounding variables of age at diagnosis, education level, gender, and race.

**Results:**

The top immediate cause of death was Alzheimer’s disease among all racial groups, except for Native American/American Indian. More females (60.3%) were affected by AD compared to males (39.7%). There is a 25% probability of survival, beyond 5 years, after AD diagnosis. Black/African American AD patients have the smallest risk of all-cause mortality across all racial/ethnic groups (HR 0.87; 95% CI, 0.85–0.89). Individuals with lower education had a lower likelihood of mortality.

**Conclusion:**

Although AD was not underreported in the state of South Carolina further research is needed to develop protocols around classification of deaths among those diagnosed with dementia and comorbidities, including cardiovascular disease, to ensure dementia is properly reported as we move to prevent and treat Alzheimer’s disease by 2025 and beyond.

## 1 Introduction

Alzheimer’s disease (AD) currently affects approximately 6.5 million Americans and is the fifth leading cause of death among those aged 65 and older. The most recent data from official death certificates recorded 121,499 deaths from AD in 2019. Given projected population growth of older adults in the coming decades, the prevalence could potentially reach 13.8 million by 2060 (Alzheimer’s Association, 2023a). Additionally, a 2023 report by the Alzheimer’s Association (2023a) reports that “5.0% of people ages 65–74, 13.1% of people 75–84 and 33.3% of people aged 85 and older” have the disease and probability estimates project these numbers are likely to increase due to population aging (Hebert et al., 2013).

Data regarding AD mortality is collected through information provided on death certificates using the International Statistical Classification of Diseases (ICD) and Related Health Problems, Tenth Revision (ICD–10) to classify and code cause of death (Kramarow and Tejada-Vera, 2018). Classification and coding assists in monitoring trends, diseases, injuries, health challenges, and interventions which may lead to informed decision-making by healthcare leaders and organizations. Classifications on death certificates are assigned by Part I or II sections. In Part I, “the disease or injury which initiated the train of morbid events immediate directly to death,” is considered *immediate*. Part II includes “any other significant condition that unfavorably influenced the course of the morbid process but is not related to the condition directly causing death,” and is therefore described as *contributing,* or *underlying*. For mortality to be attributed to AD and included in statistical reporting, AD must be listed in either Part I or Part II of the death certificate (Heron, 2016; Kramarow and Tejada-Vera, 2018; Centers for Disease Control and Prevention, 2019).

The identification of the cause of death is a matter of primary importance and a challenging issue related to overall health care and the decision-making within public health. The WHO provides guidelines for death certificates coding using the ICD based on rules for determining a single underlying cause of death and comorbidities that may have contributed to death (Kramarow and Tejada-Vera, 2018). However, ranking the causes of death did not begin in the United States until 1952 when the Public Health Conference on Records and Statistics recognized the need for a consistent national system (Centers for Disease Control and Prevention, 2019). Rankings promote better health outcomes as the selection process determines which causes of death are ranked highest or lowest. Public health experts rely on data from death certificates to identify where to allocate public health resources (Myers and Farquhar, 1998) and the National Center for Health Statistics uses the information to help identify public health challenges, determine federal and state funding, and highlight necessary research priorities (Centers for Disease Control and Prevention, 2021).

When studying mortality rates of AD, it is important to acknowledge the impact of AD reporting due to changes in ICD versions. Prior to 1994, AD was not among the list of causes available for selection for ranking on death certificates (Hoyert and Rosenberg, 1997; Della et al., 2020). Additionally, early criteria for AD diagnoses were not standardized nor assisted with laboratory and imaging options available to clinicians today. Consequently, an examination of AD mortality rates from 1979–1995 using the National Vital Statistics System demonstrated statistical differences among geographic regions and populations. Inconsistencies in reporting have been supported by studies conducted both within the United States and internationally (Kramarow and Tejada-Vera, 2018). A 2008 study comparing mortality rates indicated higher rates of AD in Puerto Rico (32.4/100,000) than that observed on the U.S. mainland (20.9/100,000) (Figueroa et al., 2008). Ganguli and Rodriguez (1999) reported that dementia was reported as the cause of death in only 58% of death certificates of individuals diagnosed with either probable AD or possible AD in Pennsylvania. In a 4 year study to measure the survival rates of pre-senile onset dementia decedents from North England, researchers used ICD-9 codes to evaluate 192 death certificates. AD was identified in 56 cases unspecified dementia was entered in 48 cases, and Alzheimer’s with cardiovascular disease in 8 cases. Heart disease was mentioned as the immediate cause of death in 27 cases and was significantly more common in those who did not have a recorded dementia of any type (Kay et al., 2000). These studies demonstrate part of the complexities involved in ascertaining causes of death and precise mortality rates associated with Alzheimer’s disease in older adults.

Other variables contributing to the complexities include education, a modifiable risk factor, which has been linked to the decreased risk of developing dementia (Dekhtyar et al., 2019). Research demonstrates that having more formal education yields ‘cognitive reserve,’ giving the brain more efficient use of cognitive neuron connections to continue carrying out cognitive daily tasks. Additionally, the higher number of completed years of education has a positive impact on health, Zajacova and Lawrence (2018) is associated with increased productivity immediate to higher earnings (i.e., socio-economic status) and the potential for more prestigious, mentally stimulating, occupations (Rogers et al., 2010) which also help create cognitive reserve (Stern et al., 2020). Other modifiable risk factors (e.g., physical activity, smoking, blood pressure, diet) may prevent or delay up to 40% of dementia cases (Livingston et al., 2020; Omura, 2022). Since brain health is directly affected by cardiovascular health, heart health is considered a contributing factor to the development of AD (Gudala et al., 2013). Between 2000 and 2019, reported deaths from AD increased more than 145% although deaths from heart disease decreased (Alzheimer’s Association, 2023a). The purpose of this descriptive study is to understand and characterize the causes of death among those who have been diagnosed with Alzheimer’s disease and related dementias (ADRD), using a comprehensive ADRD registry from South Carolina between 2014–2019.

## 2 Materials and methods

### 2.1 Data set and analytic sample

Formal consent for this study was not required. The data for this study came from the South Carolina Alzheimer’s Disease and Related Dementias Registry (SCADR), a statewide registry of South Carolina residents diagnosed with or treated for ADRD. The South Carolina Department of Health and Environmental Control approved data use (DHEC IRB.22–005). The SCADR incorporates data from multiple sources that provide administrative data on inpatient hospitalizations, mental health records, Medicaid claims, emergency department visits, memory clinic encounters, vital records, and long-term care evaluations (South Carolina Alzheimer’s Disease Registry, n.d.). Inclusion criteria for the study sample included an International Classification of Diseases, 10th revision, Clinical Modification (ICD-10-CM) coded medical record indicating AD (ICD-10-CM code G30.0-G30.9) and Senile or Pre-senile dementia and (ICD-10-CM code G30.0-G30.9) (Centers for Disease Control and Prevention, 2019). Cases underlying causes of death, education, and autopsy status were obtained from death certificates obtained by linking the SCADR with vital records. The study population was restricted to individuals diagnosed with AD at the age of 26 or later and died between January 1, 2014, and December 31, 2019 (*N* = 78,534). In Cox regression analysis, individuals with missing values in either gender, race, or educational level were excluded, which resulted in a total of 72,082 records.

### 2.2 Data variables and data collection

We utilized the ICD-10 codes to categorize various health conditions systematically (Centers for Disease Control and Prevention, 2019). Comorbid causes of death (CCB1-CCB3) were reported on death certificates, where CCB1 represents the immediate cause of death. Immediate causes of death include Alzheimer’s disease, unspecified dementia, heart disease, lung disease, stroke, pneumonia, and acute myocardial infarction, and other causes.

The primary outcome of interest was time to death (T), defined as the number of days from AD diagnosis to death. However, the nature of the data presents a unique challenge as time-to-death was interval censored, i.e., only known to fall within a certain interval rather than being known precisely. For example, if a person was diagnosed with AD in 2017 and subsequently died in 2018, the number of days survived could be any number between 1 and 729 days, i.e., 1 < =T < = 729. This is because this person might have been diagnosed with AD on December 31, 2017, and died on January 1, 2018. Conversely, it is also possible that the AD diagnosis occurred on January 1, 2017, while the individual passed away on December 31, 2018.

Sociodemographic variables considered are race (White/Caucasian, Black/African American, Asian, American Indian, Hispanic, and other races not listed), autopsy performed (yes or no), education level (8th grade or less, 9–12 grade but no diploma, high school graduate or GED (General Educational Development) completed, some college credit but no degree, associate degree, bachelor’s degree, master’s degree, and doctorate degree), and gender (female/male).

### 2.3 Data analysis

In descriptive analyses, we calculated frequencies of demographic and health-related characteristics (i.e., CCB1, age at AD diagnosis). In addition, we utilized Turnbull’s method (Turnbull, 1976) to estimate the survival probabilities or curves for different subgroups of patients as the time from AD diagnosis to death is interval censored. Lastly, we fitted the Cox proportional hazards models (Cox, 1972) and computed the hazard ratios (HRs), adjusting for the following confounding variables: age at diagnosis, education level, gender, and race.

All analyses were performed using the R software, especially the “icenReg” package (Anderson-Bergman, 2017), for fitting the Cox model with interval-censored data. Confidence intervals for the hazard ratios were derived using the delta method (Dorfman, 1938).

## 3 Results

Of 78,534 AD patients included in our study, 74,288 (94.6%) were diagnosed with AD at age 61 or older. Among this older age group, many deaths occurred in the age range between 86 and 95, with the second largest following from aged 76 to 85, highlighting the severity of the disease among the older population. Black/African American people accounted for 22.02% (17,293) of the study population. Autopsies were performed for only 1.4% of deaths. Moreover, it was observed that more females (60.3%) were affected by AD compared to males (39.7%). Most individuals were either high school graduates (37.6%) or did not have a high school degree (30.8%). Table 1 presents the comprehensive frequency distribution for each sociodemographic covariate.

**Table 1:** Social demographics frequency table.

Variable	Category	Frequency
Age at AD diagnosis	60 or less	4,246 (5.4%)
(Total:78534)	61–70	10,739 (13.7%)
	71–80	23,230 (29.6%)
	81–90	30,229 (38.5%)
	91–100	9,835 (12.5%)
	Larger than 100	255 (0.3%)
Race	White/Caucasian	53,499 (68.1%)
(Total:78534)	Black/African American	17,293 (22.0%)
	Asian	190 (0.2%)
	American Indian	62 (0.1%)
	Hispanic	240 (0.3%)
	Other than listed	1,142 (1.5%)
	Unknown/missing	6,108 (7.8%)
Autopsy	Yes	1,124 (1.4%)
(Total:78534)	No	77,307 (98.4%)
	Unknown/missing	103 (0.1%)
Gender	Male	31,206 (39.7%)
(Total:78534)	Female	47,327 (60.3%)
	Unknown/missing	1 (0.0%)
Education	8th grade or less	13,243 (16.9%)
(Total:78534)	9–12 grade but no diploma	10,934 (13.9%)
	High school graduate or GED completed	29,557 (37.6%)
	Some college credit but no degree	7,757 (9.9%)
	Associate degree	4,857 (6.2%)
	Bachelor’s degree	7,710 (9.8%)
	Master’s degree	2,909 (3.7%)
	Doctorate degree	1,168 (1.5%)
	Unknown/missing	399 (0.5%)

CCB1 signifies the immediate cause of death as recorded on the death certificate. Table 2 summarizes the top five immediate causes of death listed under CCB1 by age at AD diagnosis, and following, Table 3 presents the immediate causes of death stratified by racial categories. In Table 2 it is evident that AD and unspecified dementia consistently rank as the top two causes of death for most patients across all age groups, except for those aged 60 or younger. Notably, among patients aged ≤60, the primary cause of death shifts to C349: Malignant neoplasm of unspecified part of bronchus or lung. Furthermore, in the 61–70 age group, C349 also features among the top five causes of death.

**Table 2:** Top five causes of deaths listed in CCB1 stratified by age at AD diagnosis 2014–2019.

Age at AD diagnosis	CCB1	Frequency
60 or less	C349: Malignant neoplasm of unspecified part of bronchus or lung	178 (4.2%)
(Total:4246)	I64: Stroke	170 (4.0%)
	I219: Acute myocardial infarction	157 (3.7%)
	F03: Unspecified dementia	156 (3.7%)
	J449: Lung disease	153 (3.6%)
61–70	G309: Alzheimer’s disease	840 (7.8%)
(Total:10739)	F03: Unspecified dementia	687 (6.4%)
	J449: Lung disease	588 (5.5%)
	C349: Malignant neoplasm of unspecified part of bronchus or lung	444 (4.1%)
	I64: Stroke	413 (3.8%)
71–80	G309: Alzheimer’s disease	3,450 (14.9%)
(Total:23230)	F03: Unspecified dementia	2,542 (10.9%)
	J449: Lung disease	1,029 (4.4%)
	I251: Heart disease	963 (4.1%)
	G20: Parkinson’s disease	886 (3.8%)
81–90	G309: Alzheimer’s disease	5,471 (18.1%)
(Total:30229)	F03: Unspecified dementia	4,052 (13.4%)
	I251: Heart disease	1,325 (4.4%)
	I64: Stroke	1,050 (3.5%)
	J449: Lung disease	1,045 (3.5%)
91–100	G309: Alzheimer’s disease	1,694 (17.2%)
(Total:9835)	F03: Unspecified dementia	1,485 (15.1%)
	I251: Heart disease	449 (4.6%)
	I500: Heart failure	385 (3.9%)
	I64: Stroke	374 (3.8%)
Larger than 100	F03: Unspecified dementia	43 (16.9%)
(Total:255)	G309: Alzheimer’s disease	41 (16.1%)
	I251: Heart disease	8 (3.1%)
	I64: Stroke	7 (2.7%)
	I500: Heart failure	7 (2.7%)

**Table 3:** Top five causes of deaths listed in CCB1 stratified by race 2014–2019.

Race	CCB1	Frequency
White/Caucasian	G309: Alzheimer’s disease	8,525 (15.9%)
(Total:53499)	F03: Unspecified dementia	6,076 (11.4%)
	J449: Lung disease	2,424 (4.5%)
	I251: Heart disease	2,332 (4.4%)
	I64: Stroke	1844 (3.4%)
Black/African American	G309: Alzheimer’s disease	1816 (10.5%)
(Total:17293)	F03: Unspecified dementia	1711 (9.9%)
	I64: Stroke	754 (4.4%)
	I251: Heart disease	657 (3.8%)
	I219: Acute myocardial infarction	526 (3.0%)
Asian	G309: Alzheimer’s disease	28 (14.7%)
(Total:190)	F03: Unspecified dementia	15 (7.9%)
	I251: Heart disease	10 (5.3%)
	J189: Pneumonia	6 (3.2%)
	I48: Atrial fibrillation and flutter	6 (3.2%)
Native American/American Indian	J449: Lung disease	5 (8.1%)
(Total:62)	G309: Alzheimer’s disease	5 (8.1%)
	I64: Stroke	4 (6.5%)
	I251: Heart disease	4 (6.5%)
	G20: Parkinson’s disease	3 (4.8%)
Hispanic	G309: Alzheimer’s disease	38 (15.8%)
(Total: 240)	F03: Unspecified dementia	29 (12.1%)
	I251: Heart disease	10 (4.2%)
	I64: Stroke	8 (3.3%)
	I500: Heart failure	6 (2.5%)
Other than listed	G309: Alzheimer’s disease	187 (16.4%)
(Total:1142)	F03: Unspecified dementia	131 (11.5%)
	I64: Stroke	49 (4.3%)
	I251: Heart disease	39 (3.4%)
	I500: Heart failure	37 (3.2%)
Unknown	G309: Alzheimer’s disease	1,040 (17.0%)
(Total: 6108)	F03: Unspecified dementia	1,000 (16.4%)
	J449: Lung disease	219 (3.6%)
	I64: Stroke	209 (3.4%)
	I251: Heart disease	208 (3.4%)

Among all racial groups, except for the Native American/American Indian (NA/AI) subgroup, the top immediate cause of death was Alzheimer’s disease. In the NA/AI subgroup, lung disease tied with Alzheimer’s disease as the most frequently reported, while unspecified dementia is not in the top five. Notably, distinctive patterns emerged when analyzing specific racial groups. The third immediate cause of death among White individuals is lung disease, succeeded by heart disease and stroke. For Black and African American people, stroke ranks as the third, followed by heart disease and acute myocardial infarction. Among Asian individuals, heart disease ranked third, followed by pneumonia and atrial fibrillation/flutter but heart disease was the third major cause, succeeded by stroke and heart failure for Hispanics. For NA/AI populations, stroke and heart disease were tied as the third principal cause, followed by Parkinson’s disease.

As shown in Table 3, Alzheimer’s disease, unspecified dementia, lung disease, heart disease, and stroke are most often reported on death certificates of Alzheimer’s patients. However, when examining the differences of reported deaths from year to year (Supplementary Tables S1–S6), there are notable differences. The NA/AI group did not list Alzheimer’s disease as a top 5 cause of death until 2019 (Supplementary Table S6). Asian and Hispanic groups listed Alzheimer’s disease as the second most reported cause of death in 2017 (Supplementary Table S4) and 2018 (Supplementary Table S5), respectively. The most notable change in report is that unspecified dementia was listed first, in 2014 (Supplementary Table S1), in all racial/ethnic categories (with NA/AI as the exception) and there was a shift in 2015 (Supplementary Table S2) with Alzheimer’s disease listed first in all racial/ethnic categories with “Unknown” as the exception as shown in Supplementary Table S2.

**Figure 1:**
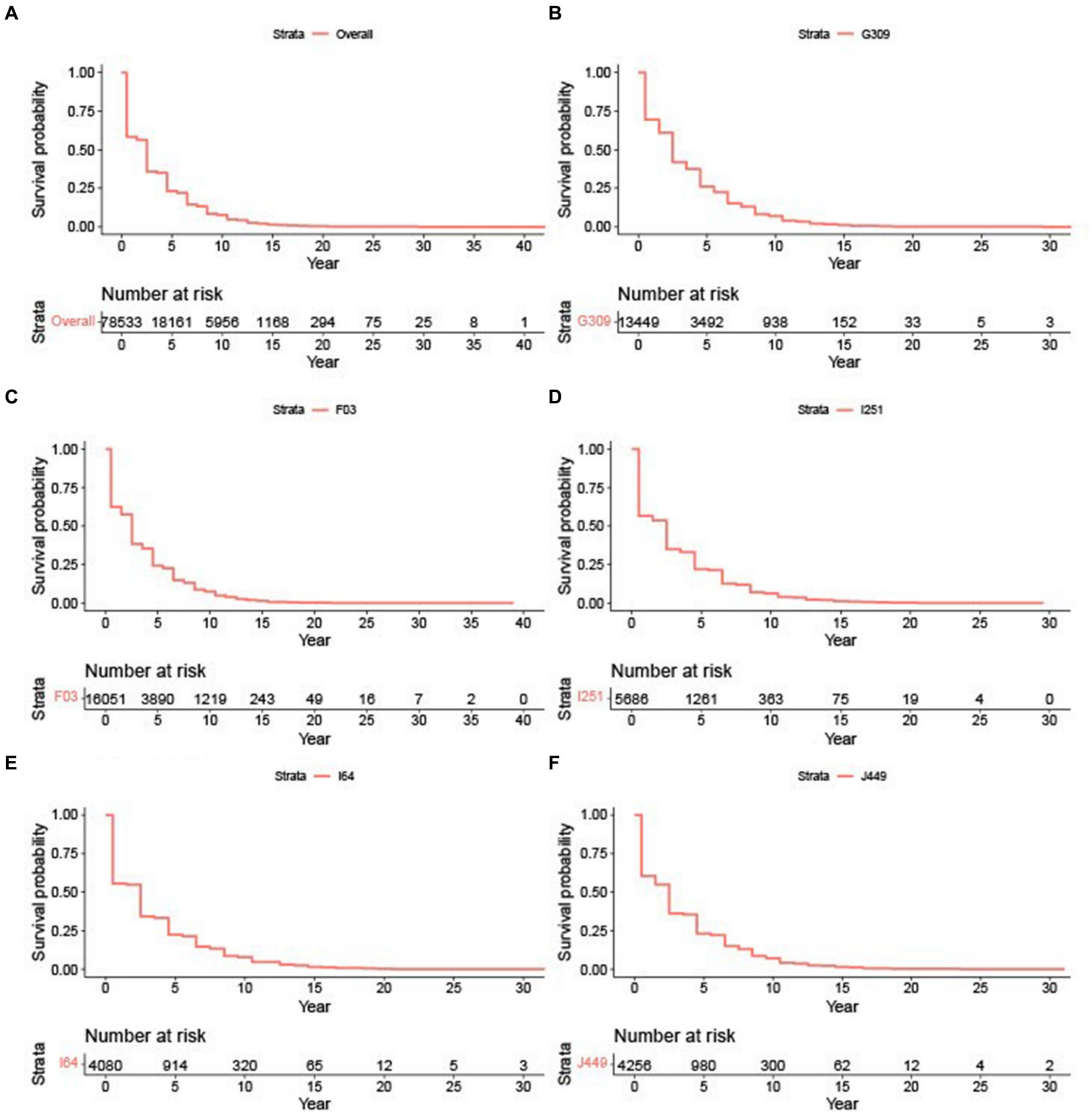
Survival plots based on cause of death. **(A)** Survival plots for all AD patients. **(B)** Survival plot for G309: Alzheimer’s disease. **(C)** Survival plot for F03: Unspecified dementia. **(D)** Survival plot for I251: Heart disease. **(E)** Survival plot for I64: Stroke. **(F)** Survival plot for J449: Lung disease.

To evaluate whether the survival experience of AD patients differs across these subgroups, we employed Turnbull’s method to estimate survival curves. Figure 1A shows the survival plot of all AD patients regardless of their causes of death. The survival probabilities drop precipitously in the first few years after diagnosis, with only a quarter of the cohort surviving beyond 5 years, but then the probabilities decrease more steadily after that. This highlights the need for effective treatments that can halt or slow down disease progression, particularly in the crucial early stages. Such pattern was consistent for Alzheimer’s disease, unspecified dementia, heart disease, stroke, and lung disease, as illustrated in Figures 1B–F. For instance, the stroke subgroup here includes AD patients with stroke listed in either CCB1, CCB2, or CCB3.

**Figure 2:**
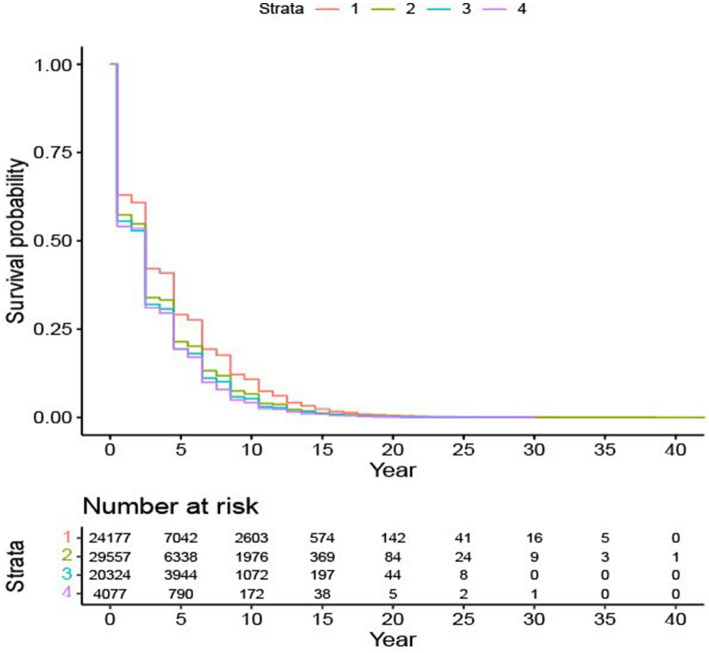
Survival probability based on education subgroups. Survival plot for four education levels: Level “1” – without a high school degree; Level “2” – graduated from high school or completed their GED certification; Level “3” – college degree; Level “4” – master’s or doctorate degree.

We consolidated educational attainment into four categories and labeled them as EDUC 1: no a high school diploma, EDUC 2: graduated from high school or completed their GED certification, EDUC 3: college degree, and EDUC 4: master’s or doctorate degree. Based on Figure 2, individuals with a lower education level had a better survival rate.

Table 4 presents the hazard ratio estimates and corresponding confidence intervals for all-cause mortality, considering the confounding variables: age at diagnosis, education level, race, and gender, using the Cox proportional hazards model. It is evident that all these variables are significantly associated with the risk of death in AD patients. The hazard ratios for those diagnosed at ages 61–70, 71–80, 81–90, 91–100, and beyond 100 are 1.49 (95% CI: 1.43, 1.55), 2.04 (95% CI: 1.97, 2.11), 2.94 (95% CI: 2.84, 3.05), 4.86 (95% CI: 4.66, 5.05), and 6.92 (95% CI: 5.80, 8.05) times higher, respectively, compared to those diagnosed at 60 or earlier.

**Table 4:** Hazard Ratios (HRs) for all-cause mortality under the Cox proportional hazards model.

Variable	HR	95% CI
Age < = 60 (ref)	-----	-----
Age 61–70	1.49	(1.43, 1.55)
Age 71–80	2.04	(1.97, 2.11)
Age 81–90	2.94	(2.84, 3.05)
Age 91–100	4.86	(4.66, 5.05)
Age > 100	6.92	(5.80, 8.05)
EDUC level 1 (ref)	-----	-----
EDUC level 2	1.17	(1.15, 1.19)
EDUC level 3	1.18	(1.16, 1.20)
EDUC level 4	1.13	(1.09, 1.17)
White/Caucasian(ref)	-----	-----
Black/African American	0.87	(0.85, 0.89)
Asian	0.98	(0.84, 1.11)
Native American/American Indian	1.08	(0.74, 1.42)
Hispanic	1.06	(0.91, 1.21)
Other than listed	0.87	(0.83, 0.91)
Female(ref)	-----	-----
Male	1.35	(1.33, 1.37)

we use the delta method to calculate the standard deviation of exp(betahat), i.e., SD(exp(betahat)) = exp(betahat) * SD(betahat). Hence, the 95% confidence interval for the hazard ratio is lower bound = exp(betahat) – 1.96 * SD(exp(betahat)) and the upper bound = exp(betahat) + 1.96 * SD(exp(betahat)).

The findings elucidated in Table 4 are consistent with the patterns delineated in Figure 2. Specifically, AD patients without a high school diploma have the lowest mortality risk, while the other three education groups are comparable. Race emerges as another crucial factor influencing the risk of death for AD patients. Among all the races considered, Black/African American AD patients have the lowest risk of all-cause mortality compared to others. Gender is also a significant determinant; male patients, as opposed to female patients, face a higher risk of death.

## 4 Discussion

This study presents an examination of death certificate reports of decedents with Alzheimer’s disease (AD) in South Carolina. Our main finding in the analysis showed that Alzheimer’s disease was listed as the first immediate cause of death, and unspecified dementia, was the second listed cause for all racial groups, except for people identified as Native American/American Indian which was tied with lung disease. Our findings contrast with previous research that reported death certificates may underreport dementia as an underlying cause of death by a factor of 2.7 in terms of percentage compared to survey-based estimates and underestimations were more likely for people identifying as Black and Hispanic (Stokes et al., 2020).

Heart disease is often listed as the number one cause of death in the United States among all deaths (Centers for Disease Control and Prevention, 2021). In our study, heart disease was present in all the racial/ethnic categories whereas it was listed third for Hispanic people and fourth for White, Black/African American, and Native American/American Indian persons. However, there was some variability in heart related diseases when considering the diagnosis of Alzheimer’s disease. Stroke was listed as a contributing cause among Black/African American and Native American/American Indian people and at a higher percentage than for White and Hispanic people. This is not a surprising finding as research on Black/African Americans people has reported this group to have higher stroke rates than other racial groups (i.e., White/Caucasian people) (Howard, 2013). The data for Native American/American Indians people, however, has not always been statistically reliable because of their small sample size and in the past, mortality demographic information has misclassified them as White. In our study, there was also a smaller sample size of NA/AI and it is unknown whether medical coroners were able to correctly identify NA/AI decedents (Harris et al., 2015). Another interesting finding was that atrial fibrillation and flutter, the immediate cause of stroke, Nesheiwat et al. (2020) was listed as the third reason for deaths among Asians but stroke was not listed in the top 5 reasons among the group.

Previous studies that have reported that level of education, including factors related to higher education (i.e., higher social economic status and healthier lifestyles), is believed to protect against the development of Alzheimer’s disease indicating those with higher levels of education were at lower risk of clinical dementia (Sharp and Gatz, 2011; Hu et al., 2022). Others provide evidence that it is not the number of educational years or attainment, but rather the intelligence one has that may serve as a confounder to the reduced risk of AD (Anderson et al., 2020). However, a study which included only probable Alzheimer’s disease diagnoses reported patients with more education had an increased mortality rate (Stern et al., 1995). Our study found that, among those with AD, individuals with lower education (i.e., no high school diploma) had a lower likelihood of mortality. While our findings align with the general understanding of Alzheimer’s disease as a progressive and degenerative condition, people with higher education may not manifest functional limitations of the disease until much later in disease progression because their education allows them to compensate.

Our ability to diagnose Alzheimer’s disease is expanded, allowing for health care professionals to better recognize the symptoms and order proper testing for diagnosis. The most notable reporting difference in the trends from 2014 to 2019 was between the years 2014 and 2015. In 2014, unspecified dementia was listed as the first immediate cause of death and in 2015 Alzheimer’s disease was the most frequently reported. This change could be attributed to the rise in action from outside entities that recognized the potential underreporting of AD among people (James et al., 2014). Death certificate information related to AD can be misinterpreted because of other common causes of death, including cardiovascular disease (Park, 2016; Stern et al., 2020). Respiratory and nervous system complications (Kershenbaum et al., 2023) are also contributing factors that often supersede AD on death certificates. At the time of the James et al. report (2014) AD was estimated as the third immediate cause of death, with dementia having a survival rate like cardiovascular disease. Additionally, the Alzheimer’s Accountability Act passed in December of 2014 for the 2015 fiscal year, requiring the National Institutes of Health to submit an annual Alzheimer’s research budget proposal directly to Congress. This budget specifies how resources may be leveraged for scientists to effectively prevent and treat Alzheimer’s disease by 2025 (Stokes et al., 2020). Finally, diagnosis of neurodegenerative disorders is typically determined through a combination of clinical assessments, bloodwork, and brain imaging such as CT, MRI, or PET scans. We suspect that the revisions to the ICD-10 which allowed for more deaths to be categorized as Alzheimer’s disease-related, increased the number of people who were identified as such on their death certificates.

There were several limitations to this study. The first limitation is not knowing the coding accuracy of the death certificates. The ICD coding system has undergone numerous iterations since the World Health Organization’s first version was released in the 1940’s. Factors contributing to the accuracy of death diagnosis and certification process include inadequate training in death certification, death certifications by clinicians/coroners who had no health or caregiving responsibilities for the decedents, varying regulations on who is permitted to act as a death certifier, the health care system (i.e., electronic health records) in which the physicians work, local coding practices (Alzheimer’s Association, 2023b).

Another limitation to the study is the potential for selection bias. Our study sample included only those with a diagnosis of dementia who died during the study period; however, many individuals die with undiagnosed dementia, especially those with earlier or more aggressive forms of the disease. Therefore, the study sample is comprised of those who survived long enough to be diagnosed and to be included in the sample, and thus may not be representative of all individuals with dementia or their causes of death. Additionally, there were smaller sample sizes for some of the racial/ethnic categories such as Native American/American Indian and Asian groups, limiting our statistical power to detect differences in mortality risk among these groups. Finally, the SCADR integrates information from a comprehensive set of sources, yet we were unable to evaluate differences according to several factors, such as existing comorbidities, socioeconomic status, health insurance, and other factors which may impact rates and/or causes of death among those with dementia.

Overall, in the case of this study, AD was not underreported in the state of South Carolina as reported in other studies (Kershenbaum et al., 2023). In fact, Alzheimer’s disease was listed as the first immediate cause of death among most racial/ethnic groups who had a diagnosis of dementia. There was a notable change in the immediate cause of death between years 2014–2015 and Alzheimer’s disease remained the immediate cause in most racial/ethnic groups through 2019. Lower education meant a lower likelihood of mortality and survival analysis indicated the significant progression at an older age potentially caused more severe cognitive and physical impairments which contributed to an elevated risk of complications and a shorter survival period following diagnosis. Further research is needed to develop protocols around classification of deaths among those diagnosed with dementia and comorbidities, including cardiovascular disease, to ensure dementia is properly reported as we move to prevent and treat Alzheimer’s disease by 2025 and beyond.

## Data availability statement

The datasets presented in this article are not readily available because the dataset was obtained from a state entity and cannot be shared without their regulated permissions. Requests to access the datasets should be directed to Jun Tang, tangj@dhec.sc.gov.

## Ethics statement

The studies involving humans were approved by South Carolina Department of Health and Environmental Control. The studies were conducted in accordance with the local legislation and institutional requirements. Written informed consent for participation was not required from the participants or the participants’ legal guardians/next of kin in accordance with the national legislation and institutional requirements.

## Author contributions

CB: Conceptualization, Funding acquisition, Investigation, Methodology, Project administration, Resources, Software, Supervision, Visualization, Writing – original draft, Writing – review & editing. XN: Formal analysis, Investigation, Methodology, Writing – original draft, Writing – review & editing. AM: Writing – original draft, Writing – review & editing. MA: Writing – original draft, Writing – review & editing. YP: Formal analysis, Investigation, Methodology, Writing – original draft, Writing – review & editing. MM: Conceptualization, Data curation, Writing – original draft, Writing – review & editing. ML: Conceptualization, Methodology, Writing – original draft, Writing – review & editing.
