# Supplementary material for: Trends in cause-specific mortality among persons with Alzheimer’s disease in South Carolina: 2014 to 2019
Source: Front Aging Neurosci. 2024 Apr 17;16:1387082. doi: 10.3389/fnagi.2024.1387082 (PMC11061437; doi:10.3389/fnagi.2024.1387082)
Supplement: Supplementary file 1 [file Table_1.DOCX]

Supplementary Material

Trends in Cause-specific Mortality among Persons with Alzheimer’s Disease in South Carolina: 2014 to 2019

Candace S. Brown^1^, Xi Ning ^2*^, Amy Money^1^, Mauriah Alford^3^, Yinghao, Pan^4^, *Margaret Miller^5^, Matthew Lohman^5^

*** Correspondence:** Corresponding Author: cbrow342@charlotte.edu

**Supplementary Table 1.**

**Top five causes of deaths listed in CCB1 stratified by race in 2014**

| **Race** | **CCB1** | **Frequency** |
| --- | --- | --- |
| White/Caucasian | F03: Unspecified dementia | 1141 (15.3%) |
| (total:7457) | G309: Alzheimer's disease | 1120 (15.0%) |
|  | I251: Heart disease | 337 (4.5%) |
|  | J449: Lung disease | 297 (4.0%) |
|  | I64: Stroke | 257 (3.4%) |
| Black/African American | F03: Unspecified dementia | 336 (12.5%) |
| (total:2692) | G309: Alzheimer's disease | 239 (8.9%) |
|  | I251: Heart disease | 123 (4.6%) |
|  | I64: Stroke | 116 (4.3%) |
|  | I219: Acute myocardial infarction | 100 (3.7%) |
| Asian | G309: Alzheimer's disease | 5 (17.2 %) |
| (total:29) | J189: Pneumonia | 2 (6.9%) |
|  | I619: Nontraumatic intracerebral hemorrhage, unspecified | 2 (6.9%) |
|  | I48: Atrial fibrillation and flutter | 2 (6.9%) |
|  | C80: Malignant neoplasm without specification of site | 2 (6.9%) |
| Native American/American Indian | J449: Lung disease | 2 (20.0%) |
| (total:10) | I64: Stroke | 2 (20.0%) |
|  | I48:Atrial fibrillation and flutter | 1 (10.0%) |
|  | I469:Cardiac arrest, cause unspecified | 1 (10.0%) |
|  | D381:Neoplasm of uncertain behavior of trachea, bronchus and lung | 1 (10.0%) |
| Hispanic | F03: Unspecified dementia | 5 (17.9%) |
| (total:28) | G309: Alzheimer's disease | 4 (14.3%) |
|  | C259: Malignant neoplasm of pancreas, unspecified | 2 (7.1%) |
|  | X590: Exposure to unspecified factor causing fracture | 1 (3.6%) |
|  | N185: Chronic kidney disease, stage 5 | 1 (3.6%) |
| Other than listed | F03: Unspecified dementia | 52 (15.6%) |
| (total:333) | G309: Alzheimer's disease | 48 (14.4%) |
|  | J449: Lung disease | 15 (4.5%) |
|  | I64: Stroke | 12 (3.6%) |
|  | I251: Heart disease | 12 (3.6%) |
| Unknown | F03: Unspecified dementia | 62 (15.0%) |
| (total: 413) | G309: Alzheimer's disease | 56 (13.6%) |
|  | I219: Acute myocardial infarction | 16 (3.9%) |
|  | I64: Stroke | 14 (3.4%) |
|  | C349: Malignant neoplasm: Bronchus or lung, unspecified | 13 (3.1%) |

**Supplementary Table 2.**

**Top five causes of deaths listed in CCB1 stratified by race in 2015**

| **Race** | **CCB1** | **Frequency** |
| --- | --- | --- |
| White/Caucasian | G309: Alzheimer's disease | 1367 (18.1%) |
| (total:7563) | F03: Unspecified dementia | 1009 (13.3%) |
|  | I251: Heart disease | 329 (4.4%) |
|  | J449: Lung disease | 303 (4.0%) |
|  | I64: Stroke | 276 (3.6%) |
| Black/African American | G309: Alzheimer's disease | 299 (11.5%) |
| (total:2590) | F03: Unspecified dementia | 297 (11.5%) |
|  | I64: Stroke | 129 (5.0%) |
|  | I219: Acute myocardial infarction | 94 (3.6%) |
|  | I251: Heart disease | 70 (2.7%) |
| Asian | G309: Alzheimer's disease | 5 (21.7 %) |
| (total:23) | F03: Unspecified dementia | 2 (8.7%) |
|  | W19: Unspecified fall | 1 (4.3%) |
|  | J690: Pneumonitis due to inhalation of food and vomit | 1 (4.3%) |
|  | J449: Lung disease | 1 (4.3%) |
| Native American/American Indian | R688: Other general symptoms and signs | 1 (20.0%) |
| (total:5) | K529: Noninfective gastroenteritis and colitis, unspecified | 1 (20.0%) |
|  | I64: Stroke | 1 (20.0%) |
|  | I251: Heart disease | 1 (20.0%) |
|  | B24: unspecified HIV disease | 1 (20.0%) |
| Hispanic | G309: Alzheimer's disease | 7 (21.2%) |
| (total:33) | I500: Heart failure | 2 (6.1%) |
|  | F03: Unspecified dementia | 2 (6.1%) |
|  | C349: Malignant neoplasm: Bronchus or lung, unspecified. | 2 (6.1%) |
|  | C220: Liver cell carcinoma | 2 (6.1%) |
| Other than listed | G309: Alzheimer's disease | 48 (23.2%) |
| (total:207) | F03: Unspecified dementia | 19 (9.2%) |
|  | I64: Stroke | 12 (5.8%) |
|  | I251: Heart disease | 11 (5.3%) |
|  | J189: Pneumonia, unspecified organism | 9 (4.3%) |
| Unknown | F03: Unspecified dementia | 59 (15.1%) |
| (total: 391) | G309: Alzheimer's disease | 52 (13.3%) |
|  | J449: Lung disease | 16 (4.1%) |
|  | I64: Stroke | 16 (4.1%) |
|  | I251: Heart disease | 14 (3.6%) |

**Supplementary Table 3.**

**Top five causes of deaths listed in CCB1 stratified by race in 2016**

| **Race** | **CCB1** | **Frequency** |
| --- | --- | --- |
| White/Caucasian | G309: Alzheimer's disease | 1571 (16.4%) |
| (total:9585) | F03: Unspecified dementia | 1102 (11.5%) |
|  | J449: Lung disease | 441 (4.6%) |
|  | I251: Heart disease | 403 (4.2%) |
|  | I64: Stroke | 343 (3.6%) |
| Black/African American | G309: Alzheimer's disease | 370 (11.8%) |
| (total:3148) | F03: Unspecified dementia | 288 (9.1%) |
|  | I251: Heart disease | 123 (3.9%) |
|  | I64: Stroke | 114 (3.6%) |
|  | I219: Acute myocardial infarction | 98 (3.1%) |
| Asian | G309: Alzheimer's disease | 8 (18.2%) |
| (total:44) | I251: Heart disease | 4 (9.1%) |
|  | I219: Acute myocardial infarction | 3 (6.8%) |
|  | F03: Unspecified dementia | 3 (6.8%) |
|  | J189: Pneumonia, unspecified organism | 2 (4.5%) |
| Native American/American Indian | F03: Unspecified dementia | 2 (16.7%) |
| (total:12) | W05: Fall from non-moving wheelchair, nonmotorized scooter and motorized mobility scooter | 1 (8.3%) |
|  | K275: Chronic or unspecified peptic ulcer, site unspecified, with perforation | 1 (8.3%) |
|  | J690: Pneumonitis due to inhalation of food and vomit | 1 (8.3%) |
|  | J441: Chronic obstructive pulmonary disease with (acute) exacerbation | 1 (8.3%) |
| Hispanic | G309: Alzheimer's disease | 8 (23.5%) |
| (total:34) | F03: Unspecified dementia | 5 (14.7%) |
|  | I694: Other mechanical complication of insulin pump | 2 (5.9%) |
|  | F019: Vascular dementia, unspecified. | 2 (5.9%) |
|  | X590: Exposure to unspecified factor causing fracture | 1 (2.9%) |
| Other than listed | G309: Alzheimer's disease | 32 (16.4%) |
| (total:195) | F03: Unspecified dementia | 22 (11.3%) |
|  | I64: Stroke | 10 (5.1%) |
|  | I219: Acute myocardial infarction | 6 (3.1%) |
|  | I500: Heart failure | 5 (2.6%) |
| Unknown | F03: Unspecified dementia | 38 (11.3%) |
| (total: 336) | G309: Alzheimer's disease | 32 (9.5%) |
|  | I251: Heart disease | 17 (5.1%) |
|  | J449: Lung disease | 16 (4.8%) |
|  | I64: Stroke | 13 (3.9%) |

**Supplementary Table 4.**

**Top five causes of deaths listed in CCB1 stratified by race in 2017**

| **Race** | **CCB1** | **Frequency** |
| --- | --- | --- |
| White/Caucasian | G309: Alzheimer's disease | 1539 (15.9%) |
| (total:9680) | F03: Unspecified dementia | 912 (9.4%) |
|  | J449: Lung disease | 475(4.9%) |
|  | I251: Heart disease | 439(4.5%) |
|  | I64: Stroke | 306(3.2%) |
| Black/African American | G309: Alzheimer's disease | 313 (10.3%) |
| (total:3037) | F03: Unspecified dementia | 268 (8.8%) |
|  | I64: Stroke | 134 (4.4%) |
|  | I251: Heart disease | 128 (4.2%) |
|  | I219: Acute myocardial infarction | 88(2.9%) |
| Asian | I251: Heart disease | 2 (8.3%) |
| (total:24) | G309: Alzheimer's disease | 2 (8.3%) |
|  | G20: Parkinson's disease without dyskinesia, without mention of fluctuations | 2 (8.3%) |
|  | F03: Unspecified dementia | 2 (8.3%) |
|  | C64: Malignant neoplasm of kidney, except renal pelvis | 2 (8.3%) |
| Native American/American Indian | I251: Heart disease | 3 (21.4%) |
| (total:14) | N288: Other specified disorders of kidney and ureter | 1 (7.1%) |
|  | N19: Unspecified kidney failure | 1 (7.1%) |
|  | J449: Lung disease | 1 (7.1%) |
|  | J439: Emphysema, unspecified | 1 (7.1%) |
| Hispanic | G309: Alzheimer's disease | 6 (12.5%) |
| (total:48) | F03: Unspecified dementia | 5 (10.4%) |
|  | J449: Lung disease | 2 (4.2%) |
|  | I500: Heart failure | 2 (4.2%) |
|  | I251: Heart disease | 2 (4.2%) |
| Other than listed | G309: Alzheimer's disease | 16 (12.1%) |
| (total:132) | F03: Unspecified dementia | 14 (10.6%) |
|  | J449: Lung disease | 5 (3.8%) |
|  | I64: Stroke | 5 (3.8%) |
|  | F019: Other psychoactive substance use, unspecified with intoxication | 5 (3.8%) |
| Unknown | G309: Alzheimer's disease | 275 (18.9%) |
| (total: 1458) | F03: Unspecified dementia | 257 (17.6%) |
|  | J449: Lung disease | 60 (4.1%) |
|  | I64: Stroke | 59 (4.0%) |
|  | I251: Heart disease | 51 (3.5%) |

**Supplementary Table 5.**

**Top five causes of deaths listed in CCB1 stratified by race in 2018**

| **Race** | **CCB1** | **Frequency** |
| --- | --- | --- |
| White/Caucasian | G309: Alzheimer's disease | 1542 (16.1%) |
| (total:9558) | F03: Unspecified dementia | 948 (9.9%) |
|  | J449: Lung disease | 442 (4.6%) |
|  | I251: Heart disease | 416 (4.4%) |
|  | I64: Stroke | 348 (3.6%) |
| Black/African American | G309: Alzheimer's disease | 316 (10.8%) |
| (total:2917) | F03: Unspecified dementia | 268 (9.2%) |
|  | I64: Stroke | 132 (4.5%) |
|  | I251: Heart disease | 112 (3.8%) |
|  | A419: Sepsis, unspecified organism | 77 (2.6%) |
| Asian | G309: Alzheimer's disease | 4 (11.4%) |
| (total:35) | F03: Unspecified dementia | 4 (11.4%) |
|  | W80: Inhalation and ingestion of other objects causing obstruction of respiratory tract | 2 (5.7%) |
|  | W19: Unspecified fall | 2 (5.7%) |
|  | I48: Atrial fibrillation and flutter | 2 (5.7%) |
| Native American/American Indian | J449: Lung disease | 2 (28.6%) |
| (total:7) | J189: Pneumonia, unspecified organism | 1 (14.3%) |
|  | I698: Sequelae of other and unspecified cerebrovascular diseases | 1 (14.3%) |
|  | I694: Cerebrovascular diseases | 1 (14.3%) |
|  | F03: Unspecified dementia | 1 (14.3%) |
| Hispanic | F03: Unspecified dementia | 7 (15.2%) |
| (total:46) | G309: Alzheimer's disease | 4 (8.7%) |
|  | I64: Stroke | 3 (6.5%) |
|  | I639: Cerebral infarction, unspecified | 2 (4.3%) |
|  | I251: Atherosclerotic heart disease of native coronary artery | 2 (4.3%) |
| Other than listed | G309: Alzheimer's disease | 25(17.7%) |
| (total:141) | F03: Unspecified dementia | 9 (6.4%) |
|  | J449: Lung disease | 7(5.0%) |
|  | I500: Heart failure | 7 (5.0%) |
|  | I251: Atherosclerotic heart disease of native coronary artery | 6 (4.3%) |
| Unknown | G309: Alzheimer's disease | 334 (19.0%) |
| (total: 1761) | F03: Unspecified dementia | 289 (16.4%) |
|  | I251: Heart disease | 62 (3.5%) |
|  | I64: Stroke | 58 (3.3%) |
|  | J449: Lung disease | 56 (3.2%) |

**Supplementary Table 6.**

**Top five causes of deaths listed in CCB1 stratified by race in 2019**

| **Race** | **CCB1** | **Frequency** |
| --- | --- | --- |
| White/Caucasian | G309: Alzheimer's disease | 1386 (14.4%) |
| (total:9656) | F03: Unspecified dementia | 964 (10.0%) |
|  | J449: Lung disease | 466 (4.8%) |
|  | I251: Heart disease | 408 (4.2%) |
|  | G20: Parkinson's disease | 325 (3.4%) |
| Black/African American | G309: Alzheimer's disease | 279 (9.6%) |
| (total:2909) | F03: Unspecified dementia | 254 (8.7%) |
|  | I64: Stroke | 129 (4.4%) |
|  | I251: Heart disease | 101 (3.5%) |
|  | I500: Heart failure | 81 (2.8%) |
| Asian | G309: Alzheimer's disease | 4 (11.4%) |
| (total:35) | F03: Unspecified dementia | 3 (8.6%) |
|  | G311: Senile degeneration of brain, not elsewhere classified | 2 (5.7%) |
|  | F019: Vascular dementia, unspecified | 2 (5.7%) |
|  | X590: Exposure to unspecified factor causing other and unspecified injury | 1 (2.9%) |
| Native American/American Indian | G309: Alzheimer's disease | 4 (28.6%) |
| (total:14) | I219: Acute myocardial infarction, unspecified | 2 (14.3%) |
|  | N288: Other specified disorders of kidney and ureter. | 1 (7.1%) |
|  | G934: Metabolic encephalopathy | 1 (7.1%) |
|  | G20: Parkinson's disease | 1 (7.1%) |
| Hispanic | G309: Alzheimer's disease | 9 (17.6%) |
| (total:51) | I251: Heart disease | 5 (9.8%) |
|  | F03: Unspecified dementia | 5 (9.8%) |
|  | J449: Lung disease | 2 (3.9%) |
|  | I698: Sequelae of other and unspecified cerebrovascular diseases | 2 (3.9%) |
| Other than listed | G309: Alzheimer's disease | 18(13.4%) |
| (total:134) | F03: Unspecified dementia | 15 (11.2%) |
|  | G311: Senile degeneration of brain, not elsewhere classified | 8 (6.0%) |
|  | I500: Heart failure | 6 (4.5%) |
|  | I64: Stroke | 5 (3.7%) |
| Unknown | F03: Unspecified dementia | 295 (16.9%) |
| (total: 1749) | G309: Alzheimer's disease | 291 (16.6%) |
|  | J449: Lung disease | 60 (3.4%) |
|  | I251: Heart disease | 54 (3.1%) |
|  | I64: Stroke | 49 (2.8%) |
